# Bulk Segregant Analysis by High-Throughput Sequencing Reveals a Novel Xylose Utilization Gene from *Saccharomyces cerevisiae*

**DOI:** 10.1371/journal.pgen.1000942

**Published:** 2010-05-13

**Authors:** Jared W. Wenger, Katja Schwartz, Gavin Sherlock

**Affiliations:** Department of Genetics, Stanford University, Stanford, California, United States of America; Cornell University, United States of America

## Abstract

Fermentation of xylose is a fundamental requirement for the efficient production of ethanol from lignocellulosic biomass sources. Although they aggressively ferment hexoses, it has long been thought that native *Saccharomyces cerevisiae* strains cannot grow fermentatively or non-fermentatively on xylose. Population surveys have uncovered a few naturally occurring strains that are weakly xylose-positive, and some *S. cerevisiae* have been genetically engineered to ferment xylose, but no strain, either natural or engineered, has yet been reported to ferment xylose as efficiently as glucose. Here, we used a medium-throughput screen to identify *Saccharomyces* strains that can increase in optical density when xylose is presented as the sole carbon source. We identified 38 strains that have this xylose utilization phenotype, including strains of *S. cerevisiae*, other *sensu stricto* members, and hybrids between them. All the *S. cerevisiae* xylose-utilizing strains we identified are wine yeasts, and for those that could produce meiotic progeny, the xylose phenotype segregates as a single gene trait. We mapped this gene by Bulk Segregant Analysis (BSA) using tiling microarrays and high-throughput sequencing. The gene is a putative xylitol dehydrogenase, which we name *XDH1*, and is located in the subtelomeric region of the right end of chromosome XV in a region not present in the S288c reference genome. We further characterized the xylose phenotype by performing gene expression microarrays and by genetically dissecting the endogenous *Saccharomyces* xylose pathway. We have demonstrated that natural *S. cerevisiae* yeasts are capable of utilizing xylose as the sole carbon source, characterized the genetic basis for this trait as well as the endogenous xylose utilization pathway, and demonstrated the feasibility of BSA using high-throughput sequencing.

## Author Summary

Ethanol made from fermentation of lignocellulosic biomass by baker's yeast can be considered “carbon neutral” and is one alternative to fossil fuels for powering vehicles. One of the recognized requirements for cost-effective and energy-efficient cellulosic ethanol production is the need to convert the sugar xylose—a major component of cellulosic biomass—into ethanol; however, it has traditionally been thought that baker's yeast cannot ferment xylose. We sought to investigate this assumption by looking at close relatives of baker's yeast from around the world to see if any had an intrinsic ability to grow on xylose. We identified a number of yeasts, many of them used in winemaking, that grow very slowly on this sugar, and studied one in detail. We determined that in this particular yeast the ability to grow on xylose is due to the presence of a single gene, which we named *XDH1*. This gene is not present in the typical laboratory strains of baker's yeast, but appears to be very common in natural wine yeasts. This gene could be useful in continuing efforts to make yeasts that can efficiently ferment xylose to ethanol.

## Introduction

It is clear that society has a responsibility to address the anthropogenic causes of climate change. Current estimates indicate that about 95% of the world's energy comes from burning fossil fuels [1], which is the leading contributor of carbon dioxide emissions. Combustion of liquid fossil fuels for transportation is responsible for a large fraction of these carbon dioxide emissions in the United States, second only to electricity generation (U.S. Environmental Protection Agency). For these reasons, creating “carbon neutral” liquid transportation fuels should be an important part of global efforts to reduce carbon emissions.

One solution already in widespread use is bioethanol fermented from sugar cane (Brazil) or cornstarch (U.S.) by various strains of *Saccharomyces cerevisiae* [2], which is used as a major component or additive to liquid transportation fuels. For bioethanol to become a sustainable, economically viable commodity, and not to compete with food sources, it is necessary to move away from sugar cane or corn biomass toward lignocellulosic biomass sources such as corn stover or other agricultural wastes, wood byproducts, or dedicated fuel crops such as *Miscanthus* or switchgrass [3]–[5]. However, there are technical challenges that must be overcome before this is possible. For sugar cane and corn biomass, the predominant sugars are glucose and/or fructose, both of which are readily fermented to ethanol by various *S. cerevisiae* yeast strains, usually wild isolates that are particularly suited for large-scale fermentations [6], [7]. However, in lignocellulosic biomass sources, the second most abundant carbohydrate after glucose is xylose, the major pentose of hemicellulose. There is as yet no known strain of *Saccharomyces* that is able to convert xylose to ethanol as efficiently as glucose. Because the mass proportion of hemicellulose ranges from 20–50% in common agricultural lignocellulosic biomasses, finding both a cost-effective and energy-efficient conversion of xylose to ethanol is a critical hurdle [8].

**Figure 1:**
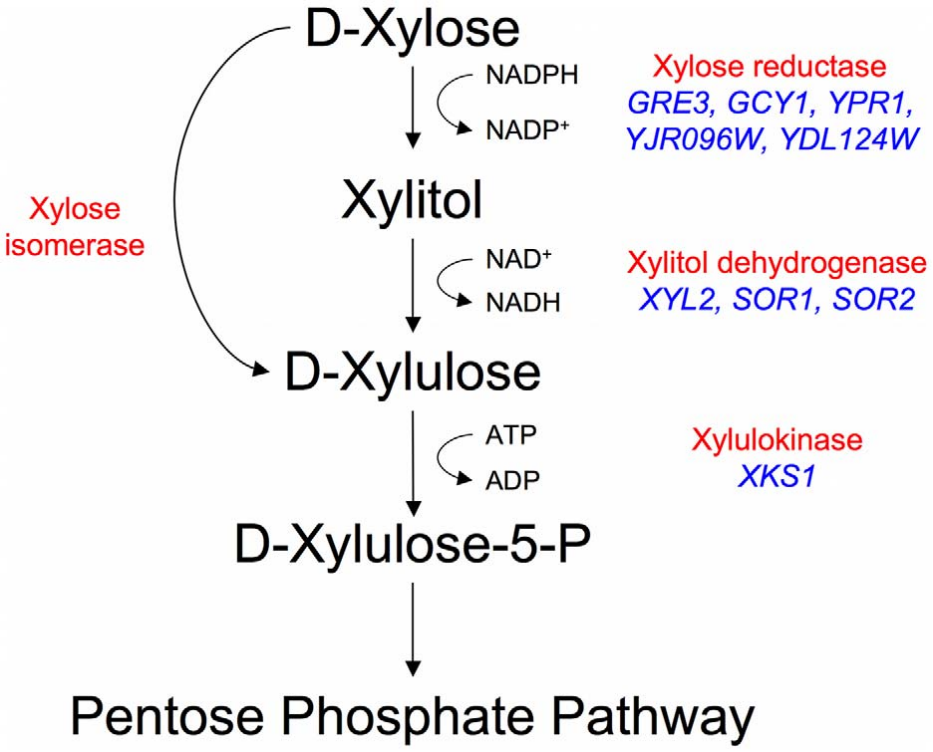
Endogenous xylose pathway. The canonical reduction-oxidation (fungi) and isomerization (bacteria and fungi) pathways with biochemical activities labeled in red. The putative *Saccharomyces cerevisiae* enzymes are in blue (there is no known xylose isomerase in *S. cerevisiae*).

The budding yeast *Saccharomyces cerevisiae* is the microorganism of choice for industrial fermentations for a variety of reasons, mainly due to its high ethanol productivity both aerobically and anaerobically, its high ethanol and low pH tolerance, and its resistance to many of the harmful compounds in a typical biomass hydrolysate. Despite recent evidence that some natural *S. cerevisiae* can grow, albeit poorly, on xylose [9], it has generally been reported that both natural and laboratory *S. cerevisiae* strains do not ferment xylose [10]–[12] leading to the assumption that they cannot, without recourse to genetic engineering, be utilized for efficient conversion of lignocellulose to ethanol. While *S. cerevisiae* strains were shown to be able to ferment the xylose isomer xylulose and to possess genes putatively encoding enzymes capable of xylose reduction (*GRE3*, *GCY1*, *YPR1*, *YDL124W*, *YJR096W*), xylitol oxidation (*XYL2*, *SOR1*, *SOR2*), and xylulose phosphorylation (*XKS1*) (Figure 1), there have been a number of experimental observations indicating that *S. cerevisiae* could not ferment xylose [13]–[15]. Such observations include low levels of gene expression of the endogenous enzymes, poor transport of xylose, redox cofactor imbalances, and insufficient flux through the pentose phosphate shunt [16], [17]. Despite these issues being well characterized in laboratory strains of *S. cerevisiae*, little is known about natural variation within *Saccharomyces* yeasts as it relates to xylose utilization which, as has already been shown [9], is likely to be relevant to this phenotype.

A significant amount of progress has been made over the last 30 years toward solving these problems, with much of the work focused on introducing foreign xylose pathway enzymes into *S. cerevisiae*: either the genes that code for xylose reductase [XR], xylitol dehydrogenase [XDH], or xylulokinase [XK] from the xylose-utilizing fungus *Pichia stipitis* [18]–[22], or genes coding for a xylose isomerase [XI] from other fungi and bacteria [23]–[28]. There have also been efforts to increase or adjust xylose pathway enzyme activities (XR, XK, XDH) [15], [29]–[33] and pentose phosphate flux [34], [35], reduce redox imbalances [36]–[41], and use directed evolution or random mutagenesis to increase xylose utilization [42]–[45]. Despite this large body of work, the fermentation of xylose to ethanol in these strains is still much slower than that of glucose, and there is still significant room for improvement in xylose fermentation, as well as co-fermentation of xylose and glucose, by *S. cerevisiae* for industrial scale applications.

As mentioned above, it has been determined that some natural strains of *Saccharomyces cerevisiae* are capable of growing on xylose, contrary to the notion that *S. cerevisiae* does not recognize this pentose as a usable carbon source [9]. It is also well characterized that there is abundant natural genetic and phenotypic variation within *S. cerevisiae* and closely related species [46]–[51]. In this work, we have screened a large number of wild, industrial and laboratory yeast strains to determine if other xylose-utilizing strains of *Saccharomyces* already exist in nature, and if so, to determine the genetic basis or bases for the phenotype. We screened 647 strains, and found a number of different *Saccharomyces* yeasts, predominantly wine yeasts, which are capable of utilizing xylose, albeit modestly. Through the application of high-throughput sequencing to Bulk Segregant Analysis [BSA] [52], we were able to identify the gene responsible for xylose utilization in a wine strain of *S. cerevisiae*, which encodes a novel putative xylitol dehydrogenase that we named *XDH1*. We observed that this gene is present in many different wine strains and is responsible for xylose utilization in these strains, however we have identified other strains in our screen that appear to have an independent genetic basis for their xylose utilization. We also carried out transcriptional profiling to characterize gene expression patterns during xylose utilization in wine strain derivatives and determined the contribution of native *S. cerevisiae* xylose pathway enzymes to the phenotype we observed. These data suggest that the putative enzyme encoded by *XDH1* works in combination with the native xylose pathway to permit natural *S. cerevisiae* strains to recognize and utilize xylose.

## Results

### Screen for Xylose Utilization

**Figure 2:**
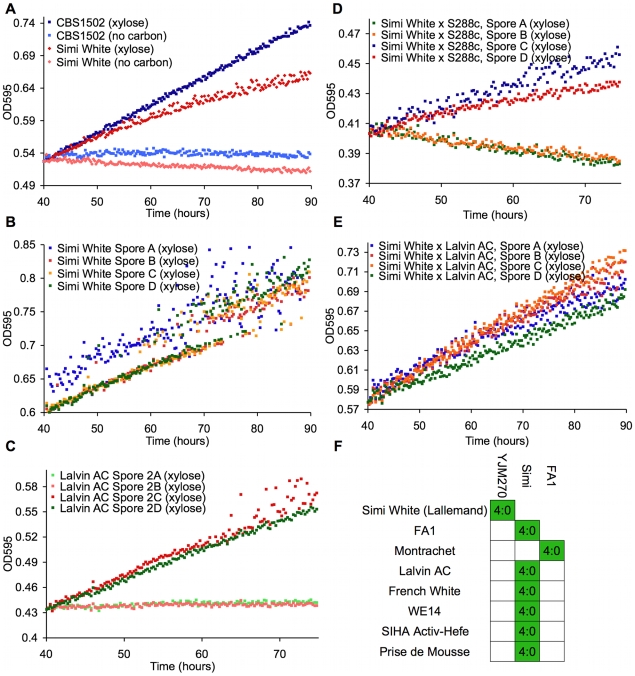
Wine strains display a xylose-utilization phenotype controlled by a single gene. These panels show growth curves measured in the TECAN. Curves are normalized to the first time point, and the initial growth phase due to trehalose (present in YP) was removed from the analysis in these and all other growth curves shown. (A) *S. cerevisiae* (Simi White) and hybrid (CBS1502) grown in YP media. (B–E) Complete tetrads of Simi White; Lalvin AC; Simi White×S288c; and Simi White×Lalvin AC. (F) This table represents all the wine strains that were able to be interbred, and all show a 4∶0 segregation of xylose utilizing∶xylose non-utilizing.

To identify natural *Saccharomyces* species/strains that are able to utilize xylose, we screened each strain in our yeast collection for the ability, when placed in liquid medium with xylose as the sole carbon source, to increase in optical density [OD] after several days of incubation at 25°C. We measured the OD of 647 strains (Table S1) in a sealed 96-well plate format with constant, orbital shaking (see Materials and Methods). The collection largely comprises *S. cerevisiae* strains from various sources, including wine, brewing, baking, laboratory and clinical isolates, but it also contains other *Saccharomyces sensu stricto* yeasts and various hybrids between them. Of the 647 strains tested, we identified 38 strains that had some observable increase in OD (Table 1). These “xylose-positive” strains were predominantly (29/38) *S. cerevisiae* wine yeasts (although not all wine yeasts were xylose-positive), with the remainder being interspecific hybrids within the *sensu stricto* group. These xylose-positive hybrid strains generally reached higher OD in xylose media compared to the *S. cerevisiae* wine strains. Figure 2A shows a typical *S. cerevisiae* wine strain profile as well as the profile from one of the best hybrids, comparing growth in a xylose-containing medium to the same medium with no carbon source. While increase in OD does not provide evidence for fermentation of xylose to ethanol, or even of cell division, these data do show that there are natural *Saccharomyces* yeasts capable of utilizing xylose to accumulate biomass.

**Table 1:** “Xylose positive” strains.

Name	Species (probable)	Origin	Rererence/Acquired	Spores*
Montrachet	*S. cerevisiae*	wine yeast from Lalvin	Vinquiry	
Montrachet	*S. cerevisiae*	wine yeast from Red Star	The Wine Lab	some −
Premier Cuvee	*S. cerevisiae*	wine yeast from Red Star	The Wine Lab	all −
UCD819	*S. cerevisiae*	Prise de Mousse wine yeast	UC Davis, Viticulture & Enology Culture Collection	some −
CC7	*S. cerevisiae/bayanus*	Y55×CBS7001	E. Louis, University of Nottingham	
CBS8614	*S. cerevisiae/bayanus/?*	cider hybrid	J. Piskur, University of Lund	
Y251	*S. cerevisiae/bayanus*	wine hybrid	J. Piskur, University of Lund	all +
G30 #2	*S. cerevisiae*	Moroccan Bread Yeast	M. Ettayebi, Sidi Mohamed Bin Abdallah University	
191-1	*S. monacensis*	Fuel ethanol yeast (Brazil)	B. Stambuk, Universidade Federal de Santa Catarina	
921 PRF21-2	*S. cerevisiae*	Dusi Ranch	Ridge Vineyards	all −
CBS424	*S. bayanus*	Switzerland	Culture Collection, Utrecht	all +
CBS1462	*S. pastorianus*	United Kingdom	Culture Collection, Utrecht	
CBS1502	*S. bayanus* or *pastorianus*	United Kingdom	Culture Collection, Utrecht	
CBS2440	*S. bayanus* or *pastorianus*	unknown	Culture Collection, Utrecht	
CBS3008	*S. bayanus*	unknown	Culture Collection, Utrecht	all +
PDM	*S. cerevisiae*	wine yeast	V. Jiranek Lab, University of Adelaide	some −
SIHA Activ-hefe 4	*S. cerevisiae*	commercial wine yeast	Begerow	some −
Fermichamp	*S. cerevisiae*	commercial wine yeast	DSM	some −
BP725	*S. cerevisiae*	commercial wine yeast	Mauri	some −
Actiflore C (F33)	*S. cerevisiae*	commercial wine yeast	Laffort	
Lalvin AC	*S. cerevisiae*	commercial wine yeast	Lallemand	2∶2 +∶−
YJM270	*S. cerevisiae*	vineyard isolate	[49]	all +
ATCC66283	*S. cerevisiae*	champagne isolate	[49]	some −
BDX Bordeaux Red	*S. cerevisiae*	commercial wine yeast	Lallemand	some −
EC1118	*S. cerevisiae*	commercial wine yeast	Lallemand	all −
FA1	*S. cerevisiae*	commercial wine yeast	Lallemand	all +
French White	*S. cerevisiae*	commercial wine yeast	Lallemand	some −
Premier Cuvee	*S. cerevisiae*	commercial wine yeast	Lesaffre	some −
Simi White	*S. cerevisiae*	commercial wine yeast	Lallemand	all +
CS2	*S. cerevisiae*	commercial wine yeast	Lallemand	
SIHA Activ-hefe 3	*S. cerevisiae*	commercial wine yeast	Begerow	all +
71B	*S. cerevisiae*	commercial wine yeast	Lallemand	
PDM	*S. cerevisiae*	commercial wine yeast	Mauri	some −
Primeur	*S. cerevisiae*	commercial wine yeast	Mauri	
Simi White	*S. cerevisiae*	commercial wine yeast	Mauri	all +
Enoferm M1	*S. cerevisiae*	commercial wine yeast	Lallemand	
Fermicru LVCB	*S. cerevisiae*	commercial wine yeast	DSM	some −
WE14	*S. cerevisiae*	commercial wine yeast	Anchor	2∶2 +∶−

*Some strains progeny were not tested because all spores were inviable, + = xylose positive, − = xylose negative.

To understand the genetic basis of this xylose utilization we chose to focus on the wine strains because many could be sporulated and crossed to a laboratory strain of *S. cerevisiae*, and we could thus determine the segregation pattern of the phenotype. Twenty-five of these xylose-positive *S. cerevisiae* strains could be sporulated and tetrads dissected (Table 1). Note that because the strains have a wild-type *HO* gene, the spore products obtained after tetrad dissection are actually fully homozygous diploids, due to self-mating of the haploid spore during its growth on the dissection plate. In 8/25 of the xylose-positive *S. cerevisiae* strains, all of the spore products were xylose-positive (e.g. Simi White, Figure 2B), while in 2/25 the trait segregated 2 xylose-positive: 2 xylose-negative (e.g. Lalvin AC, Figure 2C). In the remaining 15 strains, including three strains from which no xylose-positive spores were recovered, spore viability was so poor that no complete tetrads were obtained, and thus the segregation pattern(s) could not be identified. We then took xylose-positive spore products from all of the strains from which such spores could be obtained, and crossed them (see Materials and Methods) to a laboratory *S. cerevisiae* strain, S288c. We observed that all of the resulting diploids were xylose-positive, indicating that the phenotype is dominant (data not shown). The resulting strains were then sporulated, and in those strains where a segregation pattern could be established, the xylose-positive trait segregated to produce two positive and two negative spores, suggesting that a single gene was responsible for the xylose-positive trait (e.g. Simi White, Figure 2D). To determine if the same locus is responsible for xylose utilization in these various wine strains, we crossed xylose-positive spores between the various wine strains and determined the segregation pattern of the xylose phenotype in the progeny of these crosses. In all of the crosses that were performed, the xylose-positive phenotype segregated 4∶0 in six tetrads (Figure 2E); this defines a cohort of at least 9 wine strains containing a single complementation group (locus) responsible for the phenotype (Figure 2F). These data indicate that a single, dominant locus is responsible for permitting xylose utilization in these *S. cerevisiae* strains and suggest that this mechanism of xylose utilization is common to all of the xylose-positive wine yeasts that we identified. These data also suggest that this locus may be identical by descent, consistent with evidence that wine strains are very closely related and have probably only diverged a few thousand years ago [46], [49], [50].

### Identification of the Responsible Gene by Bulk Segregant Analysis

**Figure 3:**
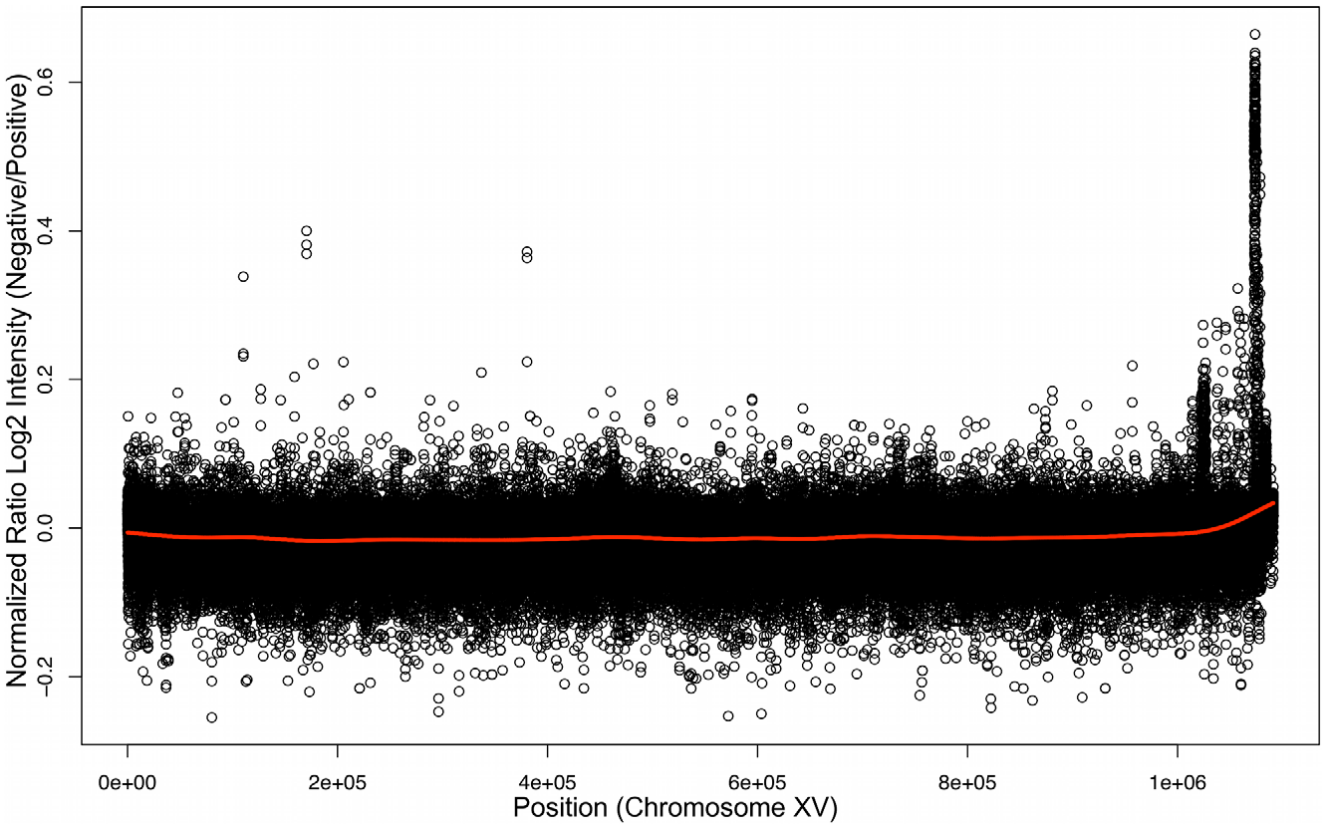
Bulk Segregant Analysis by Affymetrix Yeast tiling microarrays. Genomic DNA from pools of xylose utilizing or non-utilizing segregants were hybridized independently to Affymetrix tiling microarrays. Plotted here is a ratio of the log_2_ intensities of the xylose non-utilizing versus xylose utilizing microarray experiments along chromosome XV.

To determine the genomic location of the gene that permits xylose utilization we conducted BSA [53] using Affymetrix yeast tiling arrays. BSA works by taking advantage of DNA sequence polymorphisms between different strains and of the fact that it is relatively easy to pool large numbers of meiotic spore products (segregants) in yeast. Pooling segregants based on their phenotype allows the region of the genome responsible for the phenotype to be detected because DNA polymorphisms in regions unlinked to the responsible locus will segregate randomly and be “evened” out, while sequences or polymorphisms either directly responsible for the trait, or very closely linked to it, will be present in all positive segregants and absent in all negative segregants. In our case, the Simi White wine strain carrying the locus responsible for xylose utilization was crossed to a laboratory strain; the wine strain was previously estimated to carry DNA polymorphisms relative to the laboratory strain at a level of approximately .5% [54]. Spores from the Simi White/S288c diploid were screened for the xylose utilization phenotype and 39 positive spores were combined into one pool and 39 negative spores into another pool, and genomic DNA [gDNA] was isolated from each pool. We then hybridized the positive and negative gDNA pools to tiling microarrays (based on the S288c reference genome) with the expectation that regions of the genome derived from Simi White will hybridize less robustly to the array because of the DNA polymorphisms between Simi White and S288c. Log_2_ ratios of probe intensities were calculated (negative/positive), and a peak was evident by visual inspection in the chromosome XV right subtelomeric region that corresponds to less robust hybridization to the microarray of the positive pool gDNA (Figure 3). We confirmed the localization of the xylose-positive trait to this region by linkage analysis using strains from the yeast deletion collection, showing that the xylose-positive trait co-segregated meiotically with *PHR1* (*YOR386W*), *YOR378W*, and *YOR365C* (2). We cloned a 10 kilobase [kb] region of the genome distal to *PHR1* (containing *YOR389W*, *YOR390W*, *HSP33*, *YOR392W*, *ERR1*, and *PAU21*) from haploid, xylose-positive segregants of Simi White (GSY2469) and Lalvin AC (GSY1362) and independently transformed an S288c-based laboratory strain (FY2) with the constructs, but neither the Simi White nor the Lalvin AC derived constructs conferred a xylose-positive phenotype (data not shown), suggesting that the responsible gene was not within this 10kb region. Because yeast telomeric regions are susceptible to amplifications, insertions and translocations [55], we instead considered the possibility that the trait of interest may lie in an insertion distal to the subtelomeric sequences present in the S288c reference genome.

To identify whether there is an insertion on chromosome XV that contains the gene responsible for the xylose utilization phenotype, we repeated BSA using Illumina high-throughput sequencing on the same Simi White gDNA pools, as well as four additional pools, containing 19 positives and 16 negatives derived from a Lalvin AC/S288c cross and 16 positives and 16 negatives from a SIHA Activ-Hefe 4/S288c cross. We chose BSA over sequencing individual isolates to enrich for sequences responsible for (or tightly linked to) the xylose-positive phenotype, as there are likely to be many other novel sequences in the wine strains that are not present in the S228c genome but are unrelated to the xylose phenotype. Simi White positive and negative pools were sequenced to approximately 50× coverage of the S288c genome (∼17M mapped reads per pool), and the Lalvin AC and SIHA pools were sequenced to ∼25× coverage (∼8M mapped reads per pool) (Table S3). The 36 base pair sequence reads were aligned to the S288c reference genome using the software program MAQ [56].

**Figure 4:**
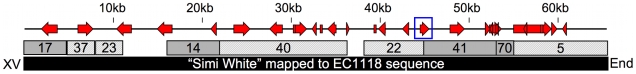
65kb insertion in subtelomeric region of chromosome XV. This map shows the positions of open reading frames within a novel chromosome XV subtelomeric region common amongst some wine strains. The blue box denotes the position of the putative xylitol dehydrogenase homolog. Numbered boxes represent Velvet contigs created from *de novo* assembly of the filtered (solid = Watson strand, hashed = Crick strand), unmapped reads compiled from the three positive pools. The black box represents the 65kb region identified by the EC1118 wine yeast genome sequence used to map the Simi White unmapped reads and find all the open reading frames in the region.

To determine if any sequences were present in the positive pool that were not present in the negative pool, we performed *de novo* assembly of the reads that did not map to the S288c reference genome. Because *de novo* assembly with short sequence reads is challenging, it is important to have deep coverage and include only high quality sequence reads. To achieve this coverage and quality, we compiled all of the high-quality unmapped reads (where “high quality” reads were defined as those that did not contain any uncalled bases) from all three positive gDNA pools and used the software program Velvet [57] to perform the assembly. We then used MAQ to independently align the unmapped reads from all six gDNA pools (positive and negative) to the Velvet contigs created from the positive pools. We identified 9 individual contigs with a combined length of approximately 55kb that had no or very few reads map to them from the three negative pools. We designed primers that would amplify each of these 9 contigs and performed linkage analysis to confirm that these contigs are linked to the xylose-positive trait and *yor365cΔ* (Table S4). We then determined that there were approximately 28 open reading frames [ORFs] (>100 amino acids) within these 9 contigs and that a number of the ORFs are homologous to sugar metabolism genes, including a xylitol/sorbitol dehydrogenase homolog (Figure 4). The presence of a large insertion relative to the S288c reference genome containing these ORFs within the right sub-telomeric region of chromosome XV has independently been recently observed in the EC1118 wine strain genome sequence [54], [58]. The total size of the insertion is 65kb, indicating that *de novo* assembly identified most of the region. These data, combined with our observation that none of the previously annotated genes in the S288c reference genome distal to *PHR1* were able to confer the xylose phenotype, strongly suggested that the xylose utilization trait resided in this telomeric insertion.

### Necessity and Sufficiency of Novel XDH Homolog

**Figure 5:**
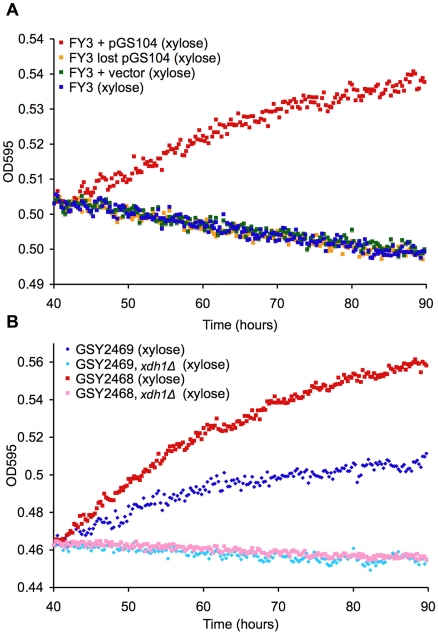
Novel XDH homolog is sufficient and necessary for xylose utilization. TECAN growth curves in YP with 2% xylose of (A) xylose-positive strain, laboratory strain transformed with pGS104 (pRS316::*XDH1*), laboratory strain transformed with pRS316 alone, and laboratory strain transformed with pGS104 but allowed to lose the plasmid and (B) two independent xylose-positive strains with an *xdh1Δ::KanMX* disruption and their parents.

Of the ORFs within the chromosome XV insertion, the putative xylitol/sorbitol dehydrogenase was particularly interesting to us because it has homology to xylitol dehydrogenases from *S. cerevisiae* and other species (Figure S1), and we hypothesized that this gene was a likely candidate for the xylose utilization trait. We amplified this gene from both Simi White and Lalvin AC, along with approximately 400 bases of upstream and downstream sequences, and cloned it into the *CEN/ARS* vector pRS316 [59] to create pGS104 and pGS105. When either of these constructs were transformed into S288c, they were sufficient to permit xylose utilization in this previously non-xylose-utilizing laboratory strain (Figure 5A and data not shown). The phenotype is dependent on the presence of the plasmid containing the gene, as the xylose phenotype was lost when the transformants lost the plasmid (Figure 5A). These data show that this gene, which we have named *XDH1*, is sufficient to permit xylose utilization in an otherwise wild type, but xylose-negative strain.

To show necessity of *XDH1* for the phenotype, we created a deletion strain (*xdh1Δ*) and measured xylose utilization as before. Two Simi White derivatives (GSY2468/9) were transformed with a *KanMX* deletion cassette containing sequences (∼400 bases) immediately up and downstream of *XDH1*. The deletion strains (GSY2472/1) were confirmed by PCR. Deletion of *XDH1* completely abrogated the phenotype (Figure 5B). We crossed the deletion strain to another haploid derivative of Simi White and confirmed that the deletion always segregates in opposition to the xylose-positive phenotype in 9 tetrads tested (data not shown). These data prove that *XDH1* is not only sufficient but also necessary for xylose utilization.

Having shown that *XDH1* is responsible for xylose utilization in at least two *S. cerevisiae* wine strains (Simi White and Lalvin AC), and also considering our observation that all the other wine strains we were able to test appeared to be in the same complementation group, we sought to determine whether *XDH1* is present in all of the xylose-positive *S. cerevisiae* strains and other *Saccharomyces* hybrids that we initially identified in our screen. To test for the presence of *XDH1* in those strains, we performed colony PCR on all of the xylose-positive strains that were identified in the screen (Table 2). In 33/38 xylose-positive isolates, *XDH1* was present. Interestingly, the 5 xylose-positive strains from which we could not amplify *XDH1* were all recorded as being either *S. bayanus* or hybrids between *S. bayanus* and *S. cerevisiae*.

**Table 2:** Presence of *XDH1* in xylose positive or negative strains.

Name	Species	Category	Xylose	*XDH1* *	*ACT1* *
Montrachet	*S. cerevisiae*	wine	+	+	+
Montrachet	*S. cerevisiae*	wine	+	+	+
Premier Cuvee	*S. cerevisiae*	wine	+	+	+
UCD819	*S. cerevisiae*	wine	+	+	+
CC7	*S. cerevisiae/bayanus*	hybrid	+	−	+
CBS8614	*S. cerevisiae/bayanus/?*	hybrid (cider)	+	−	+
Y251	*S. cerevisiae/bayanus*	hybrid (wine)	+	+	+
G30 #2	*S. cerevisiae*	baking	+	+	+
191-1	*S. monacensis*	fuel ethanol	+	+	+
921 PRF21-2	*S. cerevisiae*	wine	+	+	+
CBS424	*S. bayanus*	wild (pear juice)	+	−	+
CBS1462	*S. pastorianus*	beer	+	+	+
CBS1502	*S. bayanus* or *pastorianus*	beer	+	+	+
CBS2440	*S. bayanus* or *pastorianus*	beer	+	−	+
CBS3008	*S. bayanus*	wine	+	−	+
PDM	*S. cerevisiae*	wine	+	+	+
SIHA Activ-hefe 4	*S. cerevisiae*	wine	+	+	+
Fermichamp	*S. cerevisiae*	wine	+	+	+
BP725	*S. cerevisiae*	wine	+	+	+
Actiflore C (F33)	*S. cerevisiae*	wine	+	+	+
Lalvin AC	*S. cerevisiae*	wine	+	+	+
YJM270	*S. cerevisiae*	wine	+	+	+
ATCC66283	*S. cerevisiae*	champagne	+	+	+
BDX	*S. cerevisiae*	wine	+	+	+
EC1118	*S. cerevisiae*	wine	+	+	+
FA1	*S. cerevisiae*	wine	+	+	+
French White	*S. cerevisiae*	wine	+	+	+
Premier Cuvee	*S. cerevisiae*	wine	+	+	+
Simi White	*S. cerevisiae*	wine	+	+	+
CS2	*S. cerevisiae*	wine	+	+	+
SIHA Activ-hefe 3	*S. cerevisiae*	wine	+	+	+
71B	*S. cerevisiae*	wine	+	+	+
PDM	*S. cerevisiae*	wine	+	+	+
Primeur	*S. cerevisiae*	wine	+	+	+
Simi White	*S. cerevisiae*	wine	+	+	+
Enoferm M1	*S. cerevisiae*	wine	+	+	+
Fermicru LVCB	*S. cerevisiae*	wine	+	+	+
WE14	*S. cerevisiae*	wine	+	+	+
G17	*S. cerevisiae*	baking	−	−	+
VR1-1	*S. cerevisiae*	fuel ethanol	−	−	+
BGY	*S. cerevisiae*	wine	−	−	+
Cepage Chardonnay	*S. cerevisiae*	wine	−	−	+

*Plus indicates a PCR band observed for primers that amplify the open reading frame (*ACT1* positive control).

Some of the positive strains from our screen were heterozygous for xylose utilization, because when sporulated, the trait segregated to produce two positive and two negative spores (or some number of each type in cases where there were not enough viable spores to determine a distinct segregation pattern) (Table 1). We performed colony PCR on some of these spores to test for the presence of *XDH1* (Table 3). Among the meiotic progeny of these heterozygotes, every xylose-positive segregant contained this gene. In four cases (Lalvin AC, PDM, SIHA Activ-hefe 4, and WE14) the presence of *XDH1* segregated with the xylose-positive spores, while the negative spores did not contain *XDH1*. Surprisingly, we found instances where some negative spores did contain the *XDH1* gene. In one instance, one of the two negative spores contained *XDH1*, while the other negative spore did not (ATCC66283, note that the four spores not from the same tetrad). In the four other cases (Montrachet, BDX, Fermichamp, French White), all the negative spores tested positive by PCR for *XDH1*. We sequenced *XDH1* and approximately 200 bases up and downstream of the ORF from all spores of two of these heterozygous tetrads (Fermichamp tetrad 1A–D, BDX tetrad 1A–D) and did not observe any DNA sequence polymorphisms between the xylose-positive and negative spores (data not shown). This suggests that there may be another locus that is epistatic to *XDH1* in these strains. Overall, the ubiquity of *XDH1* in the xylose-positive strains is consistent with the hypothesis that this gene is necessary for xylose utilization in natural *S. cerevisiae* strains.

**Table 3:** Presence of *XDH1* in xylose positive progeny.

Name	Spore	Xylose	*XDH1* 1	*ACT1* 1
Montrachet (Red Star)	1B	+	+	+
Montrachet (Red Star)	1D	−	+	+
Montrachet (Red Star)	3B	+	+	+
Montrachet (Red Star)	3D	−	+	+
SIHA Activ-hefe 4	5A	+	+	+
SIHA Activ-hefe 4	5B	−	−	+
SIHA Activ-hefe 4	5C	−	−	+
SIHA Activ-hefe 4	5D	+	+	+
ATCC 66283	1B	−	+	+
ATCC 66283	2B	−	−	+
ATCC 66283	3C	+	+	+
ATCC 66283	3D	+	+	+
WE14	1A	−	−	+
WE14	1B	+	+	+
WE14	1C	+	+	+
WE14	1D	−	−	+
BDX	1A	+	+	+
BDX	1B	nd^2^	+	+
BDX	1C	−	+	+
BDX	1D	−	+	+
PDM (U Adelaide)	6A	+	+	+
PDM (U Adelaide)	7C	−	−	+
PDM (U Adelaide)	10C	+	+	+
PDM (U Adelaide)	10D	−	−	+
Fermichamp	1A	+	+	+
Fermichamp	1B	−	+	+
Fermichamp	1C	+	+	+
Fermichamp	1D	−	+	+
Lalvin AC	2A	−	−	+
Lalvin AC	2B	−	−	+
Lalvin AC	2C	+	+	+
Lalvin AC	2D	+	+	+
French White	5B	−	+	+
French White	5D	+	+	+
French White	8A	+	+	+
French White	8C	−	+	+

**1** Plus indicates a PCR band observed for primers that amplify the open reading frame (*ACT1* positive control).

**2** Not determined; strain was flocculent and unable to be scored.

### Genetic Dissection of Endogenous Xylose Pathway

**Figure 6:**
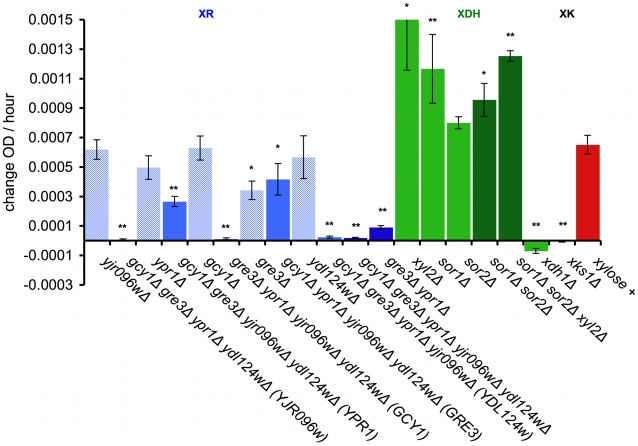
Genetic dissection of the endogenous xylose pathway. Quantification of increase in OD over time for the indicated deletions that were crossed into the Simi White haploid derivative background (GSY2469). Growth was measured in the TECAN plate reader in minimal media. OD increase calculated from slope of xylose – no carbon subtraction. * = p<.05 and ** = p<.01 in two sample t-test compared to GSY2469 (xylose +). XR = xylose reductase; XDH = xylitol dehydrogenase; XK = xylulokinase. Error bars show the standard error of the mean.

As described above, there are genes encoding putative xylose pathway enzymes in the S288c reference genome, and it has previously been suggested that the major XR contributors are *GRE3*, *YPR1*, and *YJR096W* [14]. It has also been observed that co-over-expression of *GRE3* and *XYL2*, which encodes a putative XDH, can confer a xylose-positive phenotype [14], [15]. To assess the contribution of these and the other endogenous xylose genes to our xylose phenotype, we deleted either singly or in various combinations these genes from a haploid, xylose-positive Simi White derivative (GSY2469) and assessed the growth phenotypes of the various deletion mutants (Figure 6).

To test the contribution of each of the five putative xylose reductase genes, we introduced deletions of each of them individually in the *XDH1* background. Only *GRE3* significantly affected the phenotype, and none of the xylose reductase genes, when deleted individually, completely abrogated the phenotype (Figure 6, XR). We also tested sufficiency for each of the reductases by creating quadruple deletion mutants, leaving only one putative reductase gene intact (Figure 6, XR). The only two putative xylose reductases that alone contributed significantly to the ability to utilize xylose in our background were *GRE3* and *YPR1*. The other three putative xylose reductases are insufficient by themselves to allow xylose utilization (*YDL124W*, *GCY1*, *YJR096w*). We also created a *gre3Δ ypr1Δ* double deletion in which the phenotype is almost completely removed (Figure 6, XR), though these data are not inconsistent with the other three putative xylose reductases contributing some residual XR activity. These data together suggest that both *GRE3* and *YPR1* are the major contributors to XR activity in a natural *S. cerevisiae* derivative.

Next, we tested the contribution of three putative xylitol dehydrogenases to the observed phenotype (Figure 6, XDH). Interestingly, when each potential XDH was deleted individually in the *XDH1* background (*sor1Δ*, *sor2Δ*, *xyl2Δ*), the deletion mutants showed an improved xylose utilization phenotype relative to the positive control. Furthermore, when all three were deleted together (*sor1Δ sor2Δ xyl2Δ*), the phenotype was further enhanced (Figure 6, XDH). These data suggest that these putative xylitol dehydrogenases may actually be hampering the ability of this strain (and possibly all non-xylose utilizing *S. cerevisiae* strains) to utilize xylose, and thus are consistent with our newly identified Xdh1 protein being responsible for the presumptive xylitol dehydrogenase step of the canonical xylose utilization pathway.

Finally, we introduced an *xks1Δ* deletion into the *XDH1* background, which encodes the putative xylulokinase, which is responsible for the phosphorylation of the fermentable metabolite xylulose to xylulose-5-phosphate [60], [61]. Deletion of *XKS1* completely removed the ability of this strain to utilize xylose (Figure 6, XK), suggesting that the canonical pathway in this strain is responsible for metabolizing xylose and that *XKS1* encodes the sole xylulokinase necessary for the xylose utilization phenotype we observe.

### Transcriptional Profiling during Xylose Utilization

In addition to understanding how the endogenous xylose pathway genes contribute to the xylose phenotype, we sought to characterize how the presence or absence of xylose in the growth medium affected the *S. cerevisiae* transcriptional program over time, within the genomic context of presence or absence of the *XDH1* gene. To do so, we measured mRNA levels in three pairs of sister spores from a Simi White strain that was backcrossed twice to S288c. Each pair of spores was from an independent tetrad, and contained one *XDH1*-containing spore (“positive”, GSY2465, 2466, 2469) and one spore that does not contain the *XDH1* gene (“negative”, GSY2464, 2467, 2470). We pre-grew each of the six spores in YPD and used these cultures to inoculate minimal medium with or without 2% xylose as the sole carbon source (where the absence of xylose is the “no carbon” condition). Samples were taken from these cultures beginning immediately after inoculation (t = 0) and continuing every 8 hours for 72 hours. We then assayed relative RNA abundance versus a pooled reference, containing equimolar amounts of each sample, using Agilent yeast catalog arrays. The gene expression measurements (Log_2_(sample/reference)) were averaged among the three positive spores and the three negative spores at each time point.

**Figure 7:**
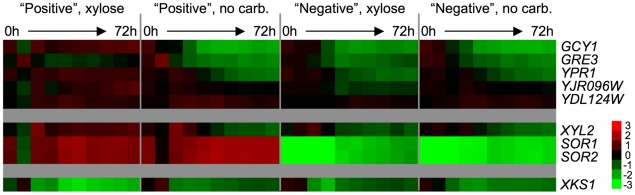
Endogenous xylose pathway gene expression. Relative mRNA abundance (compared to a pooled reference of all samples) for putative xylose pathway genes. Values are average Log_2_(sample/reference) ratios among 3 biological replicates for each time point. Time 0 is immediately following inoculation from a saturated YPD culture into “xylose” (2% xylose in minimal media) or “no carb.” (no carbon source in minimal media). Time points were taken every 8 hours for 72 hours.

To determine if the endogenous xylose pathway responds to the presence of xylose in the xylose-positive strain, we qualitatively compared the expression levels of all the putative xylose-pathway genes that are present in the *S. cerevisiae* S288c genome (Figure 7). In positive spores the putative xylose reductase genes are up-regulated compared to the reference only in the presence of xylose, while in the negative spores the xylose reductase genes are repressed under all conditions; the only exception is *YDL124W*, which appears to be up-regulated vs. the reference in all spore types and all growth conditions. The pattern of expression for the putative XDH *XYL2* is similar to that of the xylose reductase genes; it is highly expressed across the time course in the positive strain in the presence of xylose, but is repressed over the time course in the positive strain in the no carbon medium and in both the xylose and no carbon media in the negative strain.

Interestingly, the sorbitol dehydrogenases *SOR1* and *SOR2*, suggested to have the biochemical ability to oxidize xylitol, are highly expressed compared to the reference in the positive strain both in the presence and absence of xylose, and are strongly repressed vs. the pooled reference in the negative strains in both conditions across the time course. Because there is only one nucleotide difference between the coding sequences of *SOR1* and *SOR2*, the probes on the array for these genes are only different by 1 base out of 60 and thus there is likely to be cross-hybridization of the mRNA's from the two *SOR* genes. It is also possible that there is hybridization of *XDH1* mRNA to these probes, as there are only a few differences between *XDH1* and the *SOR1/2* probes on the microarray (6 for *SOR1* and 7 for *SOR2*). Although we cannot determine which of the mRNA's (*SOR1*, *SOR2* or *XDH1*) are hybridizing to the probes, it is nevertheless obvious that there is a distinct difference between the positive and negative spores in the expression levels of at least one of these putative dehydrogenase genes. No striking difference in the expression level of the xylulokinase, *XKS1*, was observed between any conditions or between any spores. The lack of change in the expression of *XKS1* is somewhat unsurprising, as it has been previously reported that low levels of *XKS1* are sufficient to allow xylose metabolism, while over-expression can enhance xylose fermentation in an engineered strain [29], [62]. Taken together, these data strongly suggest that the presence of *XDH1* in the positive spores permits continued expression of some members of the endogenous xylose pathway when grown in xylose.

**Figure 8:**
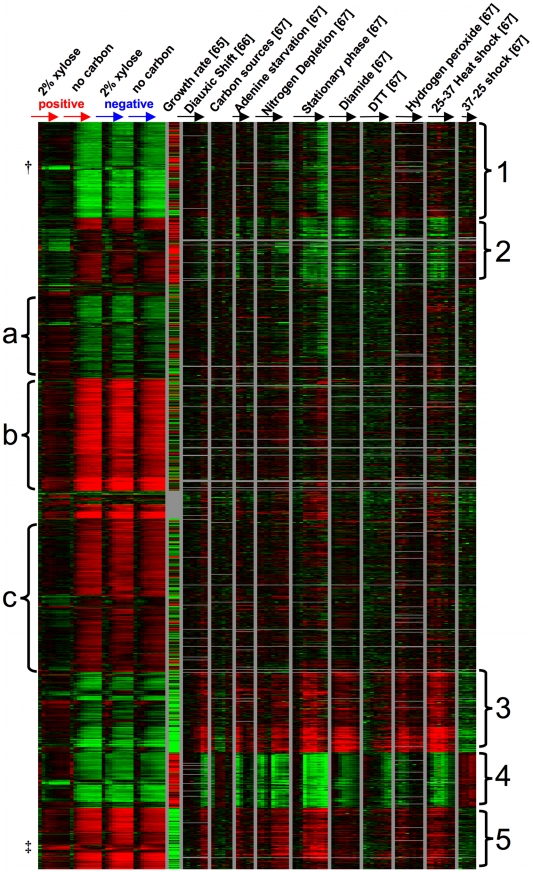
Gene expression timecourse. K-means (K = 10) clustering of gene expression values from this work and three other data sets. The 1,266 genes all changed significantly in this study in at least one two-class (paired timecourse) SAM analysis (see Results). Values from this study are time zero-transformed relative mRNA abundance (compared to a pooled reference of all samples from this work) and are averaged among 3 biological replicates at each time point. From left to right: xylose positive strain (2% xylose), xylose positive strain (no carbon source), xylose negative strain (2% xylose), xylose negative strain (no carbon source). “Growth rate” data were calculated by [65] and show the strength and direction of the transcriptional response of a given gene to a higher growth rate. “Diauxic shift” are zero-transformed data from [66], and all other data are from [67]; again all time-courses are zero-transformed. (†,‡ indicate small subgroups discussed in Results.)

To further understand the transcriptome-wide response of these strains, we identified genes that changed significantly across the time course, compared these genes with other microarray datasets to identify any clear physiological responses, and looked for categories of functional enrichment within groups of up or down-regulated genes. Using Significance Analysis of Microarrays [SAM] [63] with a false discovery rate of 1%, we identified a list of 1266 genes whose expression levels were significantly changed over time. Specifically, we carried out a SAM analysis using the two-class (paired timecourse) option to identify genes whose expression changed over time within the positive spores, comparing the xylose to the no carbon condition. Next, we identified genes whose expression changed over time when comparing the positive to the negative spores in the presence of xylose, again using SAM with a two-class (paired timecourse) option. From the union of these two gene lists, we removed genes whose expression levels changed significantly over time within the negative strain, comparing the xylose to the no carbon condition (another two-class, paired timecourse analysis). Using this strategy, we generated an inclusive list of genes whose expression values change over time due to differences between the positive and negative strain, or due to differences between the presence and absence of xylose specifically in the positive strain. To identify the physiological responses that are associated with these gene expression differences, we retrieved data for these 1266 genes using HIDRA [64] from three other yeast microarray experiments [65]–[67] and organized the genes by K-means clustering with K = 10 [68] (Figure 8, Datasets S1, S2). For consistency with the other datasets, each of the four time-course experiments performed in this work were zero-transformed. To the right of the experiments from this paper are, respectively, a measure of how each gene's expression level correlates with increased growth rate [65], a gene expression time course over the diauxic shift [66], gene expression across a set of carbon sources (ethanol, sucrose, fructose, glucose, galactose, and raffinose) [67], and a series of time courses in various conditions including starvation, steady state growth, and other stresses [67]. We observed 5 groups (labeled on the right of the heat map) that appear to be strongly driven by similarity of the positive strain in 2% xylose to either growth rate or a stress response. For example, the genes in groups 1 and 4 (Figure 8) are more highly expressed over the time course in the positive strain in xylose when compared to the positive strain in no carbon source or the negative strain in either condition, and these genes also show a positive correlation with growth rate. As expected, when GO::TermFinder [69] is used on these groups to look for functional enrichment of biological processes, we observed processes known to be up-regulated in conjunction with a higher growth rate. Specifically, group 1 was significantly enriched for vesicle-mediated transport (GO:0016192, p = 2.11e-8) and cellular localization (GO:0051641, p = 3.13e-8) among others (Dataset S3) and group 4 is enriched for translation (GO:0006412, p = 2.26e-41) and ribosome biogenesis (GO:0042254, p = 5.87e-23) along with related processes (Dataset S4). Group 5 shows the same pattern, but largely with the opposite response, meaning that these are genes whose expression is negatively correlated with growth, and we observed that they are expressed at a lower relative level in the positive strain in xylose when compared to the no carbon condition or the negative strain in either condition; but we observed no functional enrichment in this group. Interestingly, within group 5 there is a small group of genes (labeled ‡) whose expression is induced over time relative to the reference in the positive strain in xylose, and repressed over time in the other conditions. This group includes *SNO4*, *THI4*, and *HSP32*, which are genes all at least putatively involved in thiamin biosynthesis. Thiamin biosynthesis is known to be important for sugar metabolism, and is a pathway in which higher expression of certain components has likely been selected for in a variety of industrial yeasts [7]. There is also a small group of genes within group 1 (labeled †) that behaves differently than the rest of the group, as it is strongly repressed relative to the reference in the positive strain in xylose. Within this group of seven genes, four of them could be involved in intracellular redox balancing as they all use NADP(H) as a cofactor (*TRR1*, *OYE2*, *GDH1*, *ADH6*). In general, these three groups suggest that *XDH1* in the positive strain permits a “growth-like” transcriptional response in the presence of xylose, whereas in the absence of xylose or the absence of *XDH1* the strains are exhibiting an expression pattern consistent with lack of growth and starvation (e.g. groups 4 and 5). We also observed two other groups that did not fit this pattern, but instead the positive strain in xylose exhibited a response more akin to various stresses. For example, in group 2 we observed lower relative expression in the positive strain in xylose compared to the other three conditions despite the fact that these genes are all strongly correlated with growth rate, and included functional enrichment for RNA metabolism (GO:0016070, p = 1.56e-6) and ribosome biogenesis (GO:0042254, p = 1.33e-5) (Dataset S5). Instead, they appear to be more similar to the expression patterns in strains experiencing nitrogen depletion, stationary phase, diamide, DTT, or hydrogen peroxide treatment, and 37°C heat shock. We observed a similar response in group 3, in which the expression level is opposite what we might expect if growth rate was the main cause of the expression differences but similar if the strains were exhibiting an environmental stress response. Interestingly, this group was enriched for pentose metabolic process (GO:0019321, p = 5.7e-3) and response to oxidative stress (GO:0006979, p = 7.88e-3) (Dataset S6). These data suggest that despite the fact that this set of genes is normally repressed in response to a higher growth rate, some of these genes may be responding to the presence of xylose.

There were also three groups of genes that did not have an obvious visual relationship with either growth rate or stress response. Group (a) appears to be more highly expressed in the positive strain in xylose compared to no carbon or the negative strain in either condition. While this group contains no functional enrichment using GO::TermFinder, it does contain a number of genes related to carbon metabolism, including *PFK1*, *PFK2*, *PGI1*, *GCR1*, and *GND1*. The final two groups (b and c) both appear to be expressed at a lower level in the positive strain in xylose compared to the other three conditions. Both groups have functional enrichment for various processes related to transcription and its regulation (Datasets S7, S8). In general genes in these three groups (a–c) show larger magnitude expression changes (induction or repression relative to the reference) in the non-growth conditions than in the positive strain in the presence of xylose. These clusters could support the conclusion that in the absence of xylose or the absence of *XDH1*, strains are exhibiting a response (perhaps starvation) that is simply not induced in the presence of xylose in the positive strain. In summary, these microarray data suggest that the positive strain in the presence of xylose is capable of “growth” when compared to the negative strain or lack of xylose, but it is still exhibiting a less pronounced stress-like response. These data are not inconsistent with the positive strain recognizing and using xylose as a carbon source.

## Discussion

In this work we have shown that naturally occurring strains of *Saccharomyces cerevisiae* are capable of utilizing xylose without engineering or directed evolution, and have determined the genetic basis for this phenotype. While it has been known for many years that the xylose isomer xylulose is fermentable by *S. cerevisiae*, it has generally been thought that this species is incapable of metabolizing xylose. However, recent work has shown natural genetic variation for xylose utilization does exist, and that natural selection and breeding can improve xylose utilization in natural strains of *S. cerevisiae* [9]. By screening through many industrial and clinical isolates, we discovered variation within this species that permits utilization of this sugar, fermentation of which is an important prerequisite for the efficient generation of ethanol from lignocellulosic biomass sources. We have also shown that this ability to utilize xylose by *Saccharomyces* is conferred by the presence of a single gene, a novel putative xylitol dehydrogenase that we have named *XDH1*. This gene is both necessary and sufficient to permit xylose utilization in the normally non-xylose-utilizing S288c laboratory strain, and is absent from the reference genome sequence of S288c.

We also characterized the transcriptional response of one of our xylose-utilizing strains of *S. cerevisiae* to xylose in the presence and absence of *XDH1*. While these data do not allow us to draw conclusions as to whether or not this gene permits actual fermentation (rather than simply utilization) of xylose, we can make a number of observations. First, it is clear that the endogenous xylose pathway is capable of responding at the transcriptional level to the presence of xylose when this novel XDH is present. Secondly, we can infer that this sugar and its downstream metabolites are likely being funneled into central carbon metabolism via the pentose phosphate pathway as is consistent with what has previously been observed. This suggests that industrial or laboratory strains of *S. cerevisiae* may be more poised to ferment this pentose than previously thought, implying that we can better harness the standing genetic potential that already exists in nature and use it in combination with directed evolution and metabolic engineering to make an industrially applicable xylose fermentation strain.

The idea that *Saccharomyces* might be more “ready” to ferment xylose than previously thought is further supported by our genetic dissection of the xylose metabolic pathway endogenous to *S. cerevisiae*. We corroborated previous data that shows the xylulokinase encoded by *XKS1* is functional and supports metabolism of xylose. We also demonstrated that *GRE3* and *YPR1*, encoding two aldo-keto reductases, are each sufficient to allow xylose utilization in our strain background. The observation that a novel xylitol dehydrogenase is responsible for the xylose utilization phenotype, and the observation that the genes in the reference strain encoding enzymes putatively thought to oxidize xylitol (*SOR1*, *SOR2*, *XYL2*) are in fact detrimental to the phenotype, further support that the idea of a redox imbalance in *S. cerevisiae* favoring xylitol production over further metabolism is true [70], [71]. Finally, our results also suggest that some property of the *XDH1* is able to reduce the cofactor imbalance and may be capable of pushing xylitol through the xylose metabolic pathway.

We also discovered *Saccharomyces sensu stricto* interspecific hybrids in our screen that appear to robustly utilize xylose by a mechanism independent of *XDH1*. Some of these strains are even more effective at utilizing xylose than the *S. cerevisiae* wine strains we have characterized here, and we are currently attempting to identify the locus (or loci) responsible for these other xylose phenotypes. Based upon the results in Table 2 that show *S. bayanus* xylose-positive strains that do not possess *XDH1*, it is likely that there is at least one other trait that is as yet unidentified. There may also be additional components of the xylose utilization pathway for which hypomorphic alleles exist in natural strains, as *XDH1* is present in xylose-negative segregants of some xylose-positive strains we identified. We also suggest that the only other previously described [9], [72] xylose phenotype native to *S. cerevisiae* is likely to be *XDH1*-dependent, given that wine strains were included in the initial breeding. Because we and others have assayed strains that only contain a small sample of the variation that likely exists in the *Saccharomyces* gene pool, it is likely that there is additional variation present in nature that may be able to contribute to a xylose-positive phenotype.

Finally, we have developed a novel application of high-throughput sequencing for quickly mapping an unknown trait by BSA. Because we were able to identify a clear segregation pattern for our phenotype of interest, in this case a single locus, we were able to easily pool segregants and use sequencing to narrow down the genomic location using the high frequency of polymorphisms that segregated with our locus. Applying sequencing technology in addition to tiling arrays was critical as our phenotype resided in a region of the genome that is not present in the reference genome. Given that the number of genes responsible is small, we suggest that this application of high-throughput sequencing could be used broadly for associating other unknown genotypes to well-characterized phenotypes. It will be particularly applicable to other species that have small genomes and for which the genome sequence or tiling arrays are not readily available, or for such species that may contain variation not captured in their respective reference genomes.

While effective conversion of xylose to ethanol in an industrial setting by *Saccharomyces* yeasts has not yet reached its full potential, much progress has been made recently. We suggest that uncovering and studying the genes responsible for xylose utilization in wild strains of *Saccharomyces* may contribute directly to further improvements in lignocellulosic biomass fermentation. Additionally, the functions of these genes might continue to shed light on problematic areas in the metabolism of xylose, helping to inform directed evolution and metabolic engineering approaches.

## Materials and Methods

### Strains

Strains used in this study are shown in Table S1 and Table S6. In order to cross diploid *HO/HO* wine strains to a haploid S288c strain, wine strains were transformed with either pGS35 (*CEN/ARS*, *KanMX*) or pGS36 (*CEN/ARS*, *Hph*) and the resulting transformants carrying the plasmid were sporulated (*Hph* is the gene that permits hygromycin B resistance). Spores were mixed with a haploid *ho* S288c strain carrying either pGS35 if the wine strain carried pGS36 or vice versa, and plated onto YPD plates supplemented with G418 (200µg/mL) and hygromycin B (150µg/mL).

### Media, Growth Conditions, and Growth Quantification

To screen for xylose utilization, single colonies were pre-grown to saturation at 25°C in YP with 2% glucose and then diluted 1∶50 into YP with 2% xylose (Sigma) or no carbon source. 100µL cultures were grown for 5 days at 25°C in a sealed 96-well plate and absorbance was read at 595nm every 15 minutes in a TECAN Genios plate reader with orbital shaking. Xylose positives were identified by visual inspection of increasing OD in xylose compared to no carbon source, and were confirmed by retesting in both YP and Minimal [73] media.

Because growth on xylose is not exponential, we did not calculate a doubling time. Instead, to quantify xylose utilization we calculated a slope (change in OD over time) across the linear range of OD increase, from 20 to 80 hours in a typical TECAN growth experiment following the initial trehalose growth. Growth curves were done in at least triplicate (see Table S6 for all deletion strains), and a t-test was used to determine significant differences in rate of OD increase between deletion strains and “wild type” xylose positives.

### Gene Expression Arrays

To analyze gene expression, cultures were pre-grown to saturation in YP with 2% glucose and diluted 1∶50 into a 1.1L culture of minimal medium [73] with 2% xylose or no carbon source. 100mL samples were collected starting immediately after inoculation (t = 0) and at subsequent 8 hour intervals for 72 hours by filtering with 0.45µm analytical test filter funnels (Nalgene) and were snap frozen in liquid nitrogen. RNA was extracted using a modified version of the hot phenol protocol, as described [74], [75]. A pooled reference sample was created by combining 350ng of each of the 120 RNA samples (10 time points for 6 strains in 2 conditions). 325ng of each total RNA sample and reference were labeled with Cy dyes (Amersham) using the Agilent Low RNA Input Linear Amplification Kit, and hybridized to Agilent Yeast Gene Expression Arrays (v2, 8x15K) for 17 hours at 65°C at 10rpm in a hybridization oven (Shel Lab). Arrays were scanned at 5µm resolution on an Agilent Scanner, and Agilent Feature Extraction v9.5.3.1 was used for extraction of data from the scanned images, and data normalization and calculation of log_2_ ratios. Gene expression data have been deposited in the GEO database with accession number GSE19121.

### Bulk Segregant Analysis

Xylose-positive segregants of Simi White (Lallemand), Lalvin AC, and SIHA Activ-Hefe 4 were crossed once to S288c (GSY147), and the resulting diploids were then sporulated. F2 segregants were scored for xylose utilization in the TECAN plate reader as described above. 1.5mL of overnight YPD culture of each segregant grown was spun down, resuspended, and frozen in 300µL of sorbitol solution (0.9M sorbitol, 0.1M Tris pH 8, 0.1M EDTA). Samples were pooled by phenotype at this stage and genomic DNA was extracted as described [76]. The pools contained 39 positives and 39 negatives for Simi White, 19 positives and 16 negatives for Lalvin AC, and 16 positives and 16 negatives for SIHA.

Genomic DNA was labeled as described [77], and microarray-assisted BSA was done using Affymetrix GeneChip *S. cerevisiae* Tiling 1.0R Array basically as described [52], [78]. Briefly, a ratio of the log_2_ intensities for the perfect match probes was plotted across every chromosome for each nucleotide. The plots for each chromosome were scanned visually for local peaks in intensity. Tiling array data have been deposited in the GEO database with accession number GSE19121.

The same pools of genomic DNA were used for BSA by sequencing. 5µg of genomic DNA were prepared for sequencing using the Illumina Genomic DNA Sample Kit. Flow cells were prepared using the Illumina Standard Cluster Generation Kit v2, and samples were sequenced on the Illumina Genome Analyzer II. GAII data were analyzed with the Illumina 1.3.2 pipeline, and reads (with qualities) were aligned to the S288c genome with MAQ v0.7.1 [56] using default parameters. Reads from the positive pools that did not align to the reference genome were combined, and reads that contained any uncalled bases (“N”> = 1) were removed from further analysis. *De novo* assembly was performed on this filtered set of un-mapped reads using Velvet v0.7.55 [57] with default parameters and hash length = 13. All raw high throughput sequence data have been deposited in the SRA database with accession number SRP001391.

### Cloning

The novel XDH was cloned into the NotI site of pRS316 [59] from GSY2469 (Simi White derivative) and GSY1362 (Lalvin AC derivative) by PCR using primers that contained NotI restriction sites. Primers are listed in Table S5. FY2 (S288c) was then transformed with the resulting plasmids (pGS104 and pGS105) via a slightly modified lithium acetate method [79]. Plasmids are listed in Table S6. Growth was assayed as described above in the TECAN plate reader.

Plasmid loss experiments were done as follows. The original transformant that was used to generate a TECAN growth curve was also streaked for single colonies on a YPD plate. These were grown and replica plated onto YPD and SC-URA plates, and colonies were picked from the YPD plate that either retained the plasmid (grew on the SC-URA replica plate) or lost the plasmid during mitosis (did not grow on the SC-URA replica plate) and were tested again in the TECAN.

### Deletion Construction

Homologous recombination was used to create a disruption of the novel XDH. Primers are listed in Table S5. Briefly, *KanMX6* was amplified from pFA6-KanMX6 [80], and approximately 400 bases up and downstream of the XDH homolog were amplified separately using primers that overlapped with the 5′ and 3′ primers used to amplify *KanMX6*. The three fragments were joined using Phusion DNA polymerase (Finnzymes) and the resulting deletion cassette was integrated into GSY2469 and GSY2468 (Simi White derivatives) by lithium acetate transformation. Correct integration of the deletion was confirmed by PCR and by showing opposing segregation of G418 resistance and the xylose trait in a cross to another xylose-positive haploid derivative.

To genetically dissect the endogenous xylose pathway, deletions of the xylose pathway genes were crossed into a haploid Simi White derivative that was previously backcrossed twice to S288c (GSY2469). Diploid strains heterozygous for deletions of *GCY1*, *GRE3*, *YPR1*, *YJR096W*, *XYL2*, and *XKS1* were purchased from Invitrogen. Deletions of *SOR1* and *SOR2* were not available from the deletion collection as they are in large genomic regions of essentially 100% identity. Deletions were constructed as described [81], except with approximately 80 bases of homology to the regions immediately up and downstream of the *SOR1/2* open reading frames rather than 40. Transformants were crossed to *pgu1Δ* and *lrg1Δ* to differentiate between *sor1Δ* and *sor2Δ*. Segregation of deletions was tracked by colony PCR when creating strains with more than two deletions, as the deletions are all marked with G418^R^. Primers are listed in Table S5, and strains are listed in Table S6.

## Supporting Information

Dataset S1 Data from Figure 8 organized by K-means (k = 10) clustering (.cdt file).
(1.16 MB TXT)

Dataset S2 K-means groups for Figure 8 (.kgg file).
(0.01 MB TXT)

Dataset S3 GO Term Enrichment for “Group 1” from Figure 8.
(0.02 MB XLS)

Dataset S4 GO Term Enrichment for “Group 4” from Figure 8.
(0.03 MB XLS)

Dataset S5 GO Term Enrichment for “Group 2” from Figure 8.
(0.01 MB XLS)

Dataset S6 GO Term Enrichment for “Group 3” from Figure 8.
(0.01 MB XLS)

Dataset S7 GO Term Enrichment for “Group (b)” from Figure 8.
(0.01 MB XLS)

Dataset S8 GO Term Enrichment for “Group (c)” from Figure 8.
(0.07 MB XLS)

Figure S1 MUSCLE alignment of top 25 BLAST hits to *XDH1* and neighbor-joining tree based on the multiple sequence alignment.
(2.51 MB TIF)

Table S1 Strains screened for xylose utilization.
(0.09 MB XLS)

Table S2 Linkage (Tetrad) Analysis of xylose trait to various chromosome XV genes.
(0.01 MB XLS)

Table S3 Illumina Sequencing Results (using MAQ to map reads to S288c genome).
(0.02 MB XLS)

Table S4 Linkage analysis of Velvet nodes amplified by PCR to xylose positive trait (S288c *yor365c*Δ×Simi White derivative).
(0.02 MB XLS)

Table S5 Primers used in this study.
(0.04 MB XLS)

Table S6 Strains and plasmids used in this study.
(0.04 MB XLS)

## References

[1] Somerville C (2007). Biofuels.. Curr Biol.

[2] Basso LC, de Amorim HV, de Oliveira AJ, Lopes ML (2008). Yeast selection for fuel ethanol production in Brazil.. FEMS Yeast Res.

[3] Matsushika A, Inoue H, Kodaki T, Sawayama S (2009). Ethanol production from xylose in engineered *Saccharomyces cerevisiae* strains: current state and perspectives.. Appl Microbiol Biotechnol.

[4] Hahn-Hägerdal B, Galbe M, Gorwa-Grauslund MF, Liden G, Zacchi G (2006). Bio-ethanol–the fuel of tomorrow from the residues of today.. Trends Biotechnol.

[5] Farrell AE, Plevin RJ, Turner BT, Jones AD, O'Hare M (2006). Ethanol can contribute to energy and environmental goals.. Science.

[6] Argueso JL, Carazzolle MF, Mieczkowski PA, Duarte FM, Netto OV (2009). Genome structure of a *Saccharomyces cerevisiae* strain widely used in bioethanol production.. Genome Res.

[7] Stambuk B, Dunn B, Alves-Jr S, Duval E, Sherlock G (2009). Industrial Fuel Ethanol Yeasts Contain Adaptive Copy Number Changes in Genes Involved in Vitamin B1 and B6 Biosynthesis.. Genome Res.

[8] Saha BC (2003). Hemicellulose bioconversion.. J Ind Microbiol Biotechnol.

[9] Attfield PV, Bell PJL (2006). Use of population genetics to derive nonrecombinant *Saccharomyces cerevisiae* strains that grow using xylose as a sole carbon source.. FEMS Yeast Res.

[10] Chiang LC, Gong CS, Chen LF, Tsao GT (1981). d-Xylulose Fermentation to Ethanol by *Saccharomyces cerevisiae*.. Appl Environ Microbiol.

[11] Wang PY, Shopsis C, Schneider H (1980). Fermentation of a pentose by yeasts.. Biochemical and Biophysical Research Communications.

[12] Gong CS, Claypool TA, McCracken LD, Maun CM, Ueng PP (1983). Conversion of pentoses by yeasts.. Biotechnol Bioeng.

[13] Chang Q, Griest T, Harter T, Petrash J (2007). Functional studies of aldo-keto reductases in *Saccharomyces cerevisiae*.. BBA-Molecular Cell Research.

[14] Traff KL, Jonsson LJ, Hahn-Hägerdal B (2002). Putative xylose and arabinose reductases in *Saccharomyces cerevisiae*.. Yeast.

[15] Toivari MH, Salusjarvi L, Ruohonen L, Penttila M (2004). Endogenous xylose pathway in *Saccharomyces cerevisiae*.. Appl Environ Microbiol.

[16] Jeffries TW (2006). Engineering yeasts for xylose metabolism.. Curr Opin Biotechnol.

[17] Kötter P, Ciriacy M (1993). Xylose fermentation by *Saccharomyces cerevisiae*.. Appl Microbiol Biotechnol.

[18] Kötter P, Amore R, Hollenberg CP, Ciriacy M (1990). Isolation and characterization of the *Pichia stipitis* xylitol dehydrogenase gene, *XYL2*, and construction of a xylose-utilizing *Saccharomyces cerevisiae* transformant.. Curr Genet.

[19] Hallborn J, Walfridsson M, Airaksinen U, Ojamo H, Hahn-Hägerdal B (1991). Xylitol production by recombinant *Saccharomyces cerevisiae*.. Nat Biotechnol.

[20] Tantirungkij M, Nakashima N, Seki T, Yoshida T (1993). Construction of xylose-assimilating *Saccharomyces cerevisiae*.. Journal of Fermentation and Bioengineering.

[21] Ho N, Chen Z, Brainard A (1998). Genetically Engineered *Saccharomyces* Yeast Capable of Effective Cofermentation of Glucose and Xylose.. Appl Environ Microbiol.

[22] Jin YS, Jones S, Shi NQ, Jeffries TW (2002). Molecular cloning of *XYL3* (D-xylulokinase) from *Pichia stipitis* and characterization of its physiological function.. Appl Environ Microbiol.

[23] Walfridsson M, Bao X, Anderlund M, Lilius G, Bulow L (1996). Ethanolic fermentation of xylose with *Saccharomyces cerevisiae* harboring the *Thermus thermophilus xylA* gene, which expresses an active xylose (glucose) isomerase.. Appl Environ Microbiol.

[24] Amore R, Wilhelm M, Hollenberg C (1989). The fermentation of xylose – an analysis of the expression of *Bacillus* and *Actinoplanes* xylose isomerase genes in yeast.. Appl Microbiol Biotechnol.

[25] Kuyper M, Harhangi H, Stave A, Winkler A, Jetten M (2003). High-level functional expression of a fungal xylose isomerase: the key to efficient ethanolic fermentation of xylose by *Saccharomyces cerevisiae*?. FEMS Yeast Research.

[26] Kuyper M, Winkler A, Dijken J, Pronk J (2004). Minimal metabolic engineering of *Saccharomyces cerevisiae* for efficient anaerobic xylose fermentation: a proof of principle.. FEMS Yeast Research.

[27] Kuyper M, Hartog MMP, Toirkens MJ, Almering MJH, Winkler AA (2005). Metabolic engineering of a xylose-isomerase-expressing *Saccharomyces cerevisiae* strain for rapid anaerobic xylose fermentation.. FEMS Yeast Res.

[28] Madhavan A, Tamalampudi S, Ushida K, Kanai D, Katahira S (2009). Xylose isomerase from polycentric fungus *Orpinomyces*: gene sequencing, cloning, and expression in *Saccharomyces cerevisiae* for bioconversion of xylose to ethanol.. Appl Microbiol Biotechnol.

[29] Johansson B, Christensson C, Hobley T, Hahn-Hägerdal B (2001). Xylulokinase overexpression in two strains of *Saccharomyces cerevisiae* also expressing xylose reductase and xylitol dehydrogenase and its effect on fermentation of xylose and lignocellulosic hydrolysate.. Appl Environ Microbiol.

[30] Toivari MH, Aristidou A, Ruohonen L, Penttila M (2001). Conversion of xylose to ethanol by recombinant *Saccharomyces cerevisiae*: importance of xylulokinase (*XKS1*) and oxygen availability.. Metab Eng.

[31] Jin YS, Jeffries TW (2003). Changing flux of xylose metabolites by altering expression of xylose reductase and xylitol dehydrogenase in recombinant *Saccharomyces cerevisiae*.. Appl Biochem Biotechnol.

[32] Träff-Bjerre KL, Jeppsson M, Hahn-Hägerdal B, Gorwa-Grauslund M-F (2004). Endogenous NADPH-dependent aldose reductase activity influences product formation during xylose consumption in recombinant *Saccharomyces cerevisiae*.. Yeast.

[33] Jeppsson M, Traff K, Johansson Br, Hahn-Hägerdal B, Gorwa-Grauslund M (2003). Efect of enhanced xylose reductase activity on xylose consumption and product distribution in xylose-fermenting recombinant *Saccharomyces cerevisiae*.. FEMS Yeast Res.

[34] Walfridsson M, Hallborn J, Penttilä M, Keränen S, Hahn-Hägerdal B (1995). Xylose-metabolizing *Saccharomyces cerevisiae* strains overexpressing the *TKL1* and *TAL1* genes encoding the pentose phosphate pathway enzymes transketolase and transaldolase.. Appl Environ Microbiol.

[35] Jeppsson M, Johansson B, Hahn-Hägerdal B, Gorwa-Grauslund MF (2002). Reduced Oxidative Pentose Phosphate Pathway Flux in Recombinant Xylose-Utilizing *Saccharomyces cerevisiae* Strains Improves the Ethanol Yield from Xylose.. Appl Environ Microbiol.

[36] Traff KL, Otero Cordero RR, van Zyl WH, Hahn-Hägerdal B (2001). Deletion of the *GRE3* aldose reductase gene and its influence on xylose metabolism in recombinant strains of *Saccharomyces cerevisiae* expressing the *xylA* and *XKS1* genes.. Appl Environ Microbiol.

[37] Walfridsson M, Anderlund M, Bao X, Hahn-Hägerdal B (1997). Expression of different levels of enzymes from the *Pichia stipitis XYL1* and *XYL2* genes in *Saccharomyces cerevisiae* and its effects on product formation during xylose utilisation.. Appl Microbiol Biotechnol.

[38] Verho R, Londesborough J, Penttilä M, Richard P (2003). Engineering redox cofactor regeneration for improved pentose fermentation in *Saccharomyces cerevisiae*.. Appl Environ Microbiol.

[39] Petschacher B, Nidetzky B (2008). Altering the coenzyme preference of xylose reductase to favor utilization of NADH enhances ethanol yield from xylose in a metabolically engineered strain of *Saccharomyces cerevisiae*.. Microb Cell Fact.

[40] Van Vleet JH, Jeffries TW, Olsson L (2008). Deleting the para-nitrophenyl phosphatase (pNPPase), *PHO13*, in recombinant *Saccharomyces cerevisiae* improves growth and ethanol production on D-xylose.. Metabolic Engineering.

[41] Bengtsson O, Hahn-Hägerdal B, Gorwa-Grauslund MF (2009). Xylose reductase from *Pichia stipitis* with altered coenzyme preference improves ethanolic xylose fermentation by recombinant *Saccharomyces cerevisiae*.. Biotechnol Biofuels.

[42] Sonderegger M, Sauer U (2003). Evolutionary engineering of *Saccharomyces cerevisiae* for anaerobic growth on xylose.. Appl Environ Microbiol.

[43] Pitkänen J-P, Rintala E, Aristidou A, Ruohonen L, Penttilä M (2005). Xylose chemostat isolates of *Saccharomyces cerevisiae* show altered metabolite and enzyme levels compared with xylose, glucose, and ethanol metabolism of the original strain.. Appl Microbiol Biotechnol.

[44] Wahlbom CF, van Zyl WH, Jönsson LJ, Hahn-Hägerdal B, Otero RRC (2003). Generation of the improved recombinant xylose-utilizing *Saccharomyces cerevisiae* TMB 3400 by random mutagenesis and physiological comparison with *Pichia stipitis* CBS 6054.. FEMS Yeast Res.

[45] Ni H, Laplaza JM, Jeffries TW (2007). Transposon mutagenesis to improve the growth of recombinant *Saccharomyces cerevisiae* on D-xylose.. Appl Environ Microbiol.

[46] Carreto L, Eiriz MF, Gomes AC, Pereira PM, Schuller D (2008). Comparative genomics of wild type yeast strains unveils important genome diversity.. BMC Genomics.

[47] Dunn B, Levine RP, Sherlock G (2005). Microarray karyotyping of commercial wine yeast strains reveals shared, as well as unique, genomic signatures.. BMC Genomics.

[48] Kvitek DJ, Will JL, Gasch AP (2008). Variations in stress sensitivity and genomic expression in diverse *S. cerevisiae* isolates.. PLoS Genet.

[49] Fay JC, Benavides JA (2005). Evidence for domesticated and wild populations of *Saccharomyces cerevisiae*.. PLoS Genet.

[50] Liti G, Carter DM, Moses AM, Warringer J, Parts L (2009). Population genomics of domestic and wild yeasts.. Nature.

[51] Schacherer J, Shapiro JA, Ruderfer DM, Kruglyak L (2009). Comprehensive polymorphism survey elucidates population structure of *Saccharomyces cerevisiae*.. Nature.

[52] Brauer MJ, Christianson CM, Pai DA, Dunham MJ (2006). Mapping novel traits by array-assisted bulk segregant analysis in *Saccharomyces cerevisiae*.. Genetics.

[53] Quarrie S, Lazic-Jancic V, Kovacevic D, Steed A, Pekic S (1999). Bulk segregant analysis with molecular markers and its use for improving drought resistance in maize.. Journal of Experimental Botany.

[54] Borneman AR, Forgan AH, Pretorius IS, Chambers PJ (2008). Comparative genome analysis of a *Saccharomyces cerevisiae* wine strain.. FEMS Yeast Res.

[55] Louis EJ (1995). The chromosome ends of *Saccharomyces cerevisiae*.. Yeast.

[56] Li H, Ruan J, Durbin R (2008). Mapping short DNA sequencing reads and calling variants using mapping quality scores.. Genome Res.

[57] Zerbino DR, Birney E (2008). Velvet: algorithms for de novo short read assembly using de Bruijn graphs.. Genome Res.

[58] Novo M, Bigey F, Beyne E, Galeote V, Gavory F (2009). Eukaryote-to-eukaryote gene transfer events revealed by the genome sequence of the wine yeast *Saccharomyces cerevisiae* EC1118.. Proc Natl Acad Sci U S A.

[59] Sikorski R, Hieter P (1989). A System of Shuttle Vectors and Yeast Host Strains Designed for Efficient Manipulation of DNA in *Saccharomyces cerevisiae*.. Genetics.

[60] Rodriguez-Peña J, Cid V, Arroyo J (1998). The *YGR194c* (*XKS1*) gene encodes the xylulokinase from the budding yeast *Saccharomyces cerevisiae*.. FEMS Microbiology Letters.

[61] Ho N, Chang S (1989). Cloning of yeast xylulokinase gene by complementation of *E. coli* and yeast mutations.. Enzyme Microb Technol.

[62] Jin YS, Ni H, Laplaza JM, Jeffries TW (2003). Optimal growth and ethanol production from xylose by recombinant *Saccharomyces cerevisiae* require moderate D-xylulokinase activity.. Appl Environ Microbiol.

[63] Tusher VG, Tibshirani R, Chu G (2001). Significance analysis of microarrays applied to the ionizing radiation response.. Proc Natl Acad Sci U S A.

[64] Hibbs M, Wallace G, Dunham MJ, Kai L, Troyanskaya O (2007). Viewing the Larger Context of Genomic Data through Horizontal Integration..

[65] Brauer MJ, Huttenhower C, Airoldi EM, Rosenstein R, Matese JC (2008). Coordination of growth rate, cell cycle, stress response, and metabolic activity in yeast.. Mol Biol Cell.

[66] DeRisi JL, Iyer VR, Brown PO (1997). Exploring the metabolic and genetic control of gene expression on a genomic scale.. Science.

[67] Gasch AP, Spellman PT, Kao CM, Carmel-Harel O, Eisen MB (2000). Genomic expression programs in the response of yeast cells to environmental changes.. Mol Biol Cell.

[68] Everitt BS (1974). Cluster analysis.

[69] Boyle EI, Weng S, Gollub J, Jin H, Botstein D (2004). GO::TermFinder–open source software for accessing Gene Ontology information and finding significantly enriched Gene Ontology terms associated with a list of genes.. Bioinformatics.

[70] Bruinenberg P, Bot P, Dijken J, Scheffers W (1983). The role of redox balances in the anaerobic fermentation of xylose by yeasts.. Appl Microbiol Biotechnol.

[71] Jeffries T, Fiechter A, Jeffries T (1983). Utilization of xylose by bacteria, yeasts, and fungi.. Pentoses and Lignin.

[72] Bell PJ, Higgins VJ, Attfield PV (2001). Comparison of fermentative capacities of industrial baking and wild-type yeasts of the species *Saccharomyces cerevisiae* in different sugar media.. Lett Appl Microbiol.

[73] Adams J, Hansche PE (1974). Population studies in microorganisms. I. Evolution of diploidy in *Saccharomyces cerevisiae*.. Genetics.

[74] Schmitt ME, Brown TA, Trumpower BL (1990). A rapid and simple method for preparation of RNA from *Saccharomyces cerevisiae*.. Nucleic Acids Res.

[75] Lee A, Hansen KD, Bullard J, Dudoit S, Sherlock G (2008). Novel low abundance and transient RNAs in yeast revealed by tiling microarrays and ultra high-throughput sequencing are not conserved across closely related yeast species.. PLoS Genet.

[76] Treco DA, Ausubel F, Brent R, Kingston R, Moore D, Seidman J (1987). Preparation of Yeast DNA.. Curr Protoc Mol Biol.

[77] Kao KC, Sherlock G (2008). Molecular characterization of clonal interference during adaptive evolution in asexual populations of *Saccharomyces cerevisiae*.. Nat Genet.

[78] Gresham D, Ruderfer DM, Pratt SC, Schacherer J, Dunham MJ (2006). Genome-wide detection of polymorphisms at nucleotide resolution with a single DNA microarray.. Science.

[79] Schiestl RH, Gietz RD (1989). High efficiency transformation of intact yeast cells using single stranded nucleic acids as a carrier.. Curr Genet.

[80] Wach A, Brachat A, Pohlmann R, Philippsen P (1994). New heterologous modules for classical or PCR-based gene disruptions in *Saccharomyces cerevisiae*.. Yeast.

[81] Longtine MS, McKenzie A, Demarini DJ, Shah NG, Wach A (1998). Additional modules for versatile and economical PCR-based gene deletion and modification in *Saccharomyces cerevisiae*.. Yeast.

