# Supplementary figures and images for: Bulk Segregant Analysis by High-Throughput Sequencing Reveals a Novel Xylose Utilization Gene from Saccharomyces cerevisiae
Source: PLoS Genet. 2010 May 13;6(5):e1000942. doi: 10.1371/journal.pgen.1000942 (PMC2869308; doi:10.1371/journal.pgen.1000942)

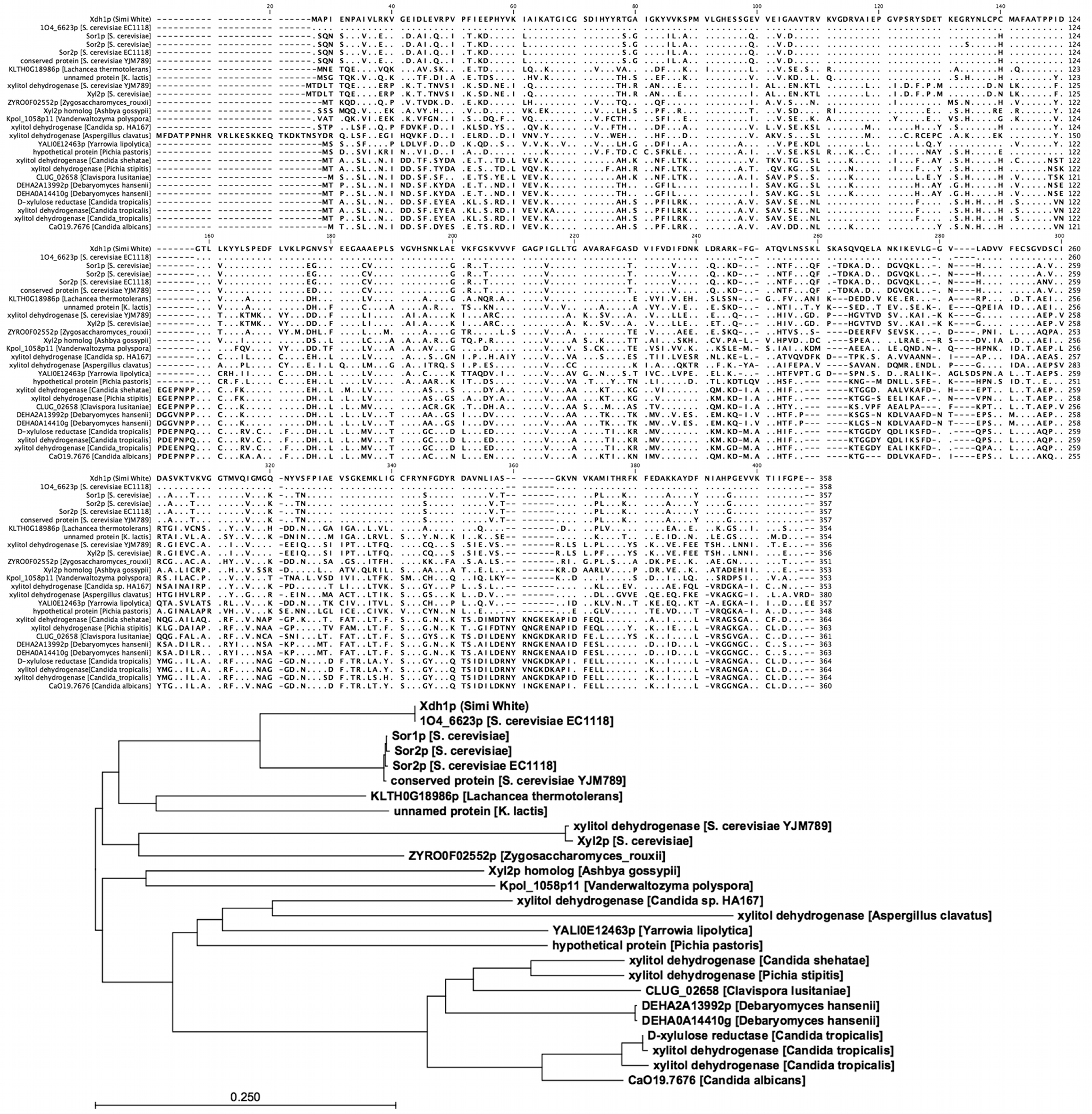

Supplement: Figure S1 — MUSCLE alignment of top 25 BLAST hits to XDH1 and neighbor-joining tree based on the multiple sequence alignment. (2.51 MB TIF) [file pgen.1000942.s009.tif]
